# The low prevalence of female smoking in the developing world: gender inequality or maternal adaptations for fetal protection?

**DOI:** 10.1093/emph/eow013

**Published:** 2016-05-18

**Authors:** Edward H. Hagen, Melissa J. Garfield, Roger J. Sullivan

**Affiliations:** ^1^Department of Anthropology, Washington State University, Vancouver, WA 98686, USA;; ^2^Department of Anthropology, California State University, Sacramento, CA 95819, USA

**Keywords:** substance use, drug toxicity, pregnancy aversions, tobacco control, global health

## Abstract

**Background:** Female smoking prevalence is dramatically lower in developing countries (3.1%) than developed countries (17.2%), whereas male smoking is similar (32% vs 30.1%). Low female smoking has been linked to high gender inequality. Alternatively, to protect their offspring from teratogenic substances, pregnant and lactating women appear to have evolved aversions to toxic plant substances like nicotine, which are reinforced by cultural proscriptions. Higher total fertility rates (TFRs) in developing countries could therefore explain their lower prevalence of female smoking.

**Objective:** To compare the associations of TFR and gender inequality with national prevalence rates of female and male smoking.

**Methods:** Data from a previous study of smoking prevalence vs gender inequality in 74 countries were reanalysed with a regression model that also included TFR. We replicated this analysis with three additional measures of gender equality and 2012 smoking data from 173 countries.

**Results:** A 1 SD increase in TFR predicted a decrease in female smoking prevalence by factors of 0.58–0.77, adjusting for covariates. TFR had a smaller and unexpected negative association with male smoking prevalence. Increased gender equality was associated with increased female smoking prevalence, and, unexpectedly, with decreased male smoking prevalence. TFR was also associated with an increase in smoking prevalence among postmenopausal women.

**Conclusions:** High TFR and gender inequality both predict reduced prevalence of female smoking across nations. In countries with high TFR, adaptations and cultural norms that protect fetuses from plant toxins might suppress smoking among frequently pregnant and lactating women.

## INTRODUCTION

*Preventing an epidemic of tobacco-related diseases among women in the developing world presents one of the greatest public health opportunities of our time.* Samet and Yoon (2010, p. 59).

**Figure 1.**
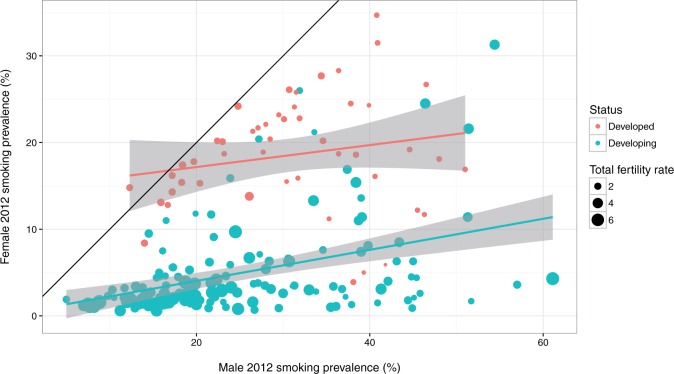
Cross-national variation in female vs male smoking prevalence in 2012 according to national developmental status. Each dot is one country. Black diagonal line represents equal smoking prevalence. Regression lines fit by ordinary least squares; bands are 95% CI. Smoking prevalence data and development status from Ref. [1]. TFRs for 2005–10 from Ref. [2]

There is little difference in mean adult male smoking prevalence in developed vs developing countries (30.1% vs 32%, respectively) [1]. Adult female smoking prevalence however, varies greatly: in more developed, westernized countries the mean female smoking prevalence is 17.2%, whereas in developing nations it is 3.1% [1], see Fig. 1. Women in the developing world are therefore the largest potential new market for tobacco products. A substantial increase in smoking in this segment of the population would dramatically increase the global burden of smoking-related disease.

Most literature on gender differences in smoking within populations has focused on differences in traditional sex roles, which historically translated into social norms, such as disapproval of female smoking on the one hand, and, on the other hand, to gender-specific personal characteristics, such as greater rebelliousness among men that increase male smoking rates [3]. Similarly, cross-national variation in female smoking prevalence is often tied to cross-national variation in women’s social, political and economic power, that is, to variation in gender inequality. In developing countries, women, on average, experience lower degrees of autonomy and have less economic and political power than in developing counties [4]. These restrictions tend to be stronger for younger women than older women [5]. Consequently, women in developing countries might face considerable social and economic barriers to obtaining and smoking cigarettes, especially younger women, whereas women in developed countries face fewer such barriers, regardless of age [6].

Thus, in countries with high gender inequality, smoking prevalence should be lower in women vs men, and in younger vs older women. In countries with low gender inequality, there should be smaller gender and age differences in smoking prevalence [6]. We term this the ‘gender inequality model’ of sex and age differences in substance use across populations.

## ADAPTATIONS FOR FETAL PROTECTION MIGHT REDUCE FEMALE DRUG USE

Alternatively, adaptations for fetal protection might reduce women’s drug use. Hook [7] and Profet [8] argued that pregnant women evolved to avoid and expel toxic plants because these pose a risk to the developing fetus, especially during organogenesis (for review, see Ref. [9]). Nicotine, caffeine and most other globally popular recreational drugs are plant defensive chemicals or their close chemical analogs (alcohol is the primary exception) [10], and there is clear evidence that nicotine is a teratogen [11].

Sullivan *et al.* [12] and Hagen *et al.* [13] argued that theories of human substance use must consider that hominin and primate ancestors were regularly exposed to similar compounds in their diets, which largely comprised wild plants, and have therefore co-evolved a number of defenses against them. Nicotine triggers most known human toxin defense mechanisms, for instance, including bitter taste receptors in the mouth and gut, bitter taste pathways in the peripheral nervous system, xenobiotic-sensing nuclear receptors, xenobiotic-metabolizing enzymes, aversion circuitry in the central nervous system and conditioned taste avoidance (reviewed in Ref. [14]). Nicotine and other drugs taste bitter, and high bitter sensitivity generally predicts reduced drug intake and might be protective against nicotine dependence [15–19].

Hagen *et al.* [14] argued that the global male bias in drug use might be explained by maternal adaptations to protect the fetus from teratogens. Sex differences in drug use would be lower in the pre-reproductive years (adolescence). During the reproductive years (∼18–40), women’s drug use would diminish relative to men’s. Post menopause, women's drug use would again converge with men’s. The evidence that, compared with men, reproductive age women have heightened innate aversions to ingesting teratogenic substances to avoid harming their fetuses and nursing infants includes that women have more fungiform papillae and more taste buds than men; are able to detect lower concentrations of bitter substances, according to most studies; and are more likely to be supertasters. Most studies have also found that women have a 20–30% higher clearance rate of drugs metabolized by *CYP3A* (a major xenobiotic metabolizing enzyme), which indicates heightened toxin defenses relative to men; for review, see Ref. [14].

Toxin defenses appear to be up-regulated during pregnancy. Approximately 50–90% of human pregnancies involve heightened food aversions, and up to 80% involve nausea and vomiting, which are associated with positive pregnancy outcomes, suggesting they function to protect the fetus from toxins and/or pathogens [7–9]. During pregnancy, expression levels of P450 genes *CYP3A4*, *CYP2C9* and *CYP2D6*, which produce enzymes that probably evolved to metabolize plant toxins and other xenobiotics, and together are responsible for the metabolism of >80% of commercial drugs, are increased several-fold. (Pregnancy-related changes in activities of other P450 enzymes are equivocal, with some evidence for increased activity of *CYP2B6*.) Pregnancy hormones are potential modulators of P450 gene expression. *In vitro*, pregnancy-levels of estradiol enhanced *CYP2A6*, *CYP2B6* and *CYP3A4* expression, whereas progesterone induced *CYP2A6, CYP2B6, CYP2C8, CYP3A4* and *CYP3A5* expression [20]. There is also evidence for increased activity of the phase II metabolic enzyme *UGT1A4*, as well as the drug transporters p-glycoprotein, *OATP1B1* and *OCT2*. For review of pregnancy drug metabolism, see Ref. [21].

Pregnancy-related dietary aversions include toxic plant drugs, such as tobacco and coffee [9, 22], and these aversions appear to reduce drug intake. Women smokers often reduce or cease smoking during pregnancy, for example, and one important reason seems to be sensory aversions to tobacco smoke [23]. Among women smokers who stop smoking during pregnancy, breastfeeding is associated with reduced risk of postpartum smoking relapse [24].

Culturally evolved norms also regulate women’s diet during pregnancy with the aim of protecting the fetus from toxic foods. In Fiji, culturally acquired pregnancy food taboos appear to protect pregnant and lactating women from the most toxic marine species [25]. In south India, culturally acquired food avoidances are aimed at protecting pregnant women from ‘hot’ foods, which are thought to cause miscarriages. ‘Hot’ foods are often fruits, such as papaya, that contain allergenic latexes and other defensive compounds, and are known abortifacients [26, 27]. In both populations, women learn which foods to avoid predominantly from their grandmothers, mothers and mothers-in-law [25–27], and in south India, at least, these proscriptions include tobacco (Placek and Hagen, in preparation) [27].

Hence, women who are frequently pregnant, that is, have high fertility, might avoid tobacco more than women with low fertility, and breastfeeding and cultural norms might augment this effect. Total fertility rates (TFRs) range from ∼1.2 in South Korea to ∼7 in Niger, Somalia and Chad [28]. Cross-national variation in TFR is closely related to the demographic or fertility transition that has been transforming the global population for the last 200 years. In most regions, the significant reductions in child mortality rates brought on by improvements in sanitation and public health were followed by population growth and subsequent reductions in fertility rates, which might have been further reduced by modern contraceptive technologies [29]. In populations with high TFR, women are pregnant or lactating for roughly the first half of their adult lives. In Afghanistan, for example, the TFR is 6.3, and over 90% of mothers are breastfeeding at 1 year and over 50% are breastfeeding at 2 years [30]. Thus, from about ages 18–35, a substantial fraction of Afghan women are either pregnant or lactating.

It is possible, then, that in countries with a high TFR, evolved fetal protection mechanisms and cultural pregnancy dietary proscriptions cause many more women to avoid teratogenic substances like tobacco. This could explain the low prevalence of female smoking in developing countries, which, on average, have significantly higher mean TFR compared to developed countries (*M* = 3.58 vs *M* = 1.61, respectively; *t* = −20, *P* = 1.27 × 10^−^^58^). Women in developed counties, on the other hand, who have low fertility and direct means of controlling their own fertility through the use of oral contraceptives, do not face the same reproductive risks when they consume tobacco, which could help explain the higher prevalence of female smoking, see Fig. 1.

On this view, cross-national variation in female smoking prevalence is determined, in part, by cross-national variation in the TFR. Smoking prevalence should also be lower in premenopausal vs postmenopausal adult women, which would explain the unique age-related increase in female smoking noted by Ref. [1]. We term this the ‘fetal protection model’ of sex and age differences in plant substance use across populations [14], see Supplementary Figs S1 and S2.

Here, using cross-national data sets, we evaluate the gender inequality model and the fetal protection model of female smoking prevalence. The two hypotheses are not mutually exclusive, and both could contribute to global smoking patterns.

## METHODS

We compared the fetal protection vs gender inequality models of female smoking using cross-national TFR and four measures of gender inequality in economic, political and social domains as explanatory variables, and smoking prevalence as the outcome variable.

The gender inequality and fetal protection models both predict that their respective explanatory variables (gender inequality indices and TFR) will be more strongly associated with female than with male smoking prevalence. To formally test these hypothesized sex differences, and to estimate their sign and magnitude, each statistical model included both female and male smoking prevalence, with ‘sex × gender inequality and sex × TFR’ interaction terms. The value and statistical significance of these interaction terms then served to test the sex difference hypotheses.

To maximize comparability with previous empirical results, we closely followed the analysis of Hitchman and Fong [6], who computed a gender smoking ratio (GSR) using cross-national female and male smoking prevalence rates from the *WHO Report on the Global Tobacco Epidemic, 2008*. They then computed a multiple regression model of GSR as a function of the gender empowerment measure (GEM), controlling for the Gini coefficient of income inequality (GINI) and log gross national income per capita (GNI) for 2008. They found that, for the 74 countries with complete data, the GEM was a significant positive predictor of the GSR.

In our first study, we repeated Hitchman and Fong’s analysis using their data, and then extended it in the following ways. Because an increase in GSR could be due to an increase in the numerator (female smoking prevalence) or a decrease in the denominator (male smoking prevalence), or both, we modeled smoking prevalence as an interaction of sex and GEM, controlling for GINI and log GNI. This allowed us to formally test whether the GEM affected female and male smoking prevalence differently, and if so, to estimate the magnitude and sign of the difference. Following the recommendations of Ref. [31], we log transformed smoking prevalence because it is positive interval data whose clinical importance relates to a ratio scale. That is, given the low prevalence of female smoking in the developing world, a change from 3% to 6% would represent a doubling, whereas a change in male smoking from 30% to 33% would represent a proportionately much smaller increase. The log transform allowed us to approximate the ratio scale of the original analysis in Ref. [6] while at the same time testing interactions with sex. We then added TFR and its interaction with sex to Hitchman and Fong’s model, to determine if it was associated with female and/or male smoking prevalence after controlling for the GEM, GINI and log GNI.

In our second study, we attempted to replicate our findings using a newer smoking data set for 2012 that included 187 countries [1]; for this, we used updated GNI values. We also included three additional measures of gender inequality, which included values for more countries than the GEM (see Table 1).

**Table 1.** Cross-national measures of gender inequality used in this study

Variable	Definition	Source	Number of countries	Scoring
GEM	**Gender Empowerment Measure** Economic participation and decision-making, political participation, and decision-making and power over economic resources	[35]	109	0–1
GGGI	**Global Gender Gap Index** Economic participation and opportunity, educational attainment, political empowerment, and health and survival	[36]	142	0–1
WECON	**Women’s Economic Rights** Laws concerning women’s economic rights	[37]	202	0–3
WOPOL	**Women’s Political Rights** Laws pertaining women’s political rights	[37]	202	0–3

All variables have a potential minimum value of 0, representing extreme gender inequality. Maximum values represent complete gender equality. Number of countries represents the original data, and not the overlap with other data sets in this study.

In our third study, we analysed smoking prevalence in postmenopausal women and older men as functions of our four measures of gender inequality, TFR and their interactions with sex, controlling for log GNI and smoking prevalence in premenopausal women and younger men.

### Outcome variable: smoking prevalence

Hitchman and Fong’s study [6] used WHO 2008 smoking data that were based on the most recent survey data for each country, which ranged from 1991 to 2007. Data were then age-adjusted to reflect the prevalence of current smoking any tobacco product among people over 15 years of age. Current smoking was defined as smoking at the time of the survey, including daily and non-daily smoking [32].

Ng *et al.* [1] provide age-adjusted smoking prevalence by age and gender for 187 countries for each year from 1980 to 2012, which to our knowledge is the most comprehensive cross-national smoking prevalence data set currently available. Ng *et al.* systematically identified and synthesized nationally representative sources that measured tobacco use including Demographic and Health Surveys, Global Youth Tobacco Surveys, Global Adult Tobacco Surveys, the World Health Organization (WHO) STEPwise Approach to Surveillance program, the Eurobarometer Surveys, the Living Standards Measurement Studies, the Multiple Indicator Cluster Surveys, the World Health Surveys and the Reproductive Health Surveys. Observations were synthesized using spatial-temporal Gaussian process regression to model prevalence estimates by age, sex, country and year. A ‘smoker’ was someone who smokes any type of tobacco product at least once per day. We used the 2012 age-standardized smoking prevalence rates for the population aged 15 years or older, which were computed using the WHO age standard [1].

We also created two smoking variables using age-specific prevalence rates from Ng *et al.* The premenopause and younger men smoking prevalence for each country was the average smoking prevalences among 20–35 year olds; the postmenopause and older men smoking prevalence for each country was the average smoking prevalence among 45–60 year olds.

### Explanatory variables

#### Measures of gender inequality

Gender disparities play an increasingly important role in both academic research and policy discussions, which has lead to a proliferation of indices that aggregate multiple indicators of gender differences at the nation level. Unfortunately, no consensus has been reached on the ideal index, with most indices facing criticism ranging from their theoretical conceptions of gender inequality to technical issues regarding measurement and validity [33]. We therefore examined four popular gender inequality indices (we deliberately exclude one popular index, the Human Development Gender Inequality Index [34], which incorporates the adolescent birth rate, and is thus confounded with TFR).

The GEM [35], used by Hitchman and Fong, had values for 109 countries. The GEM measures gender inequality in economic participation and decision-making, political participation, and decision-making and power over economic resources. Specifically, the GEM takes into account: seats in parliament held by women (percent of total); female legislators, senior officials and managers (percent of total); female professional and technical workers (percent of total); ratio of estimated female to male earned income (percent of total); the year women received right to vote and stand for election; year a woman became Presiding Officer of parliament or of one of its houses for the first time; and percent of women in ministerial positions. Low scores indicate high gender inequality, and high scores indicate low gender inequality.

The Global Gender Gap Index (GGGI) [36], which is available for 142 countries, categorizes gender equality indicators along four dimensions: economic participation and opportunity, educational attainment, political empowerment, and health and survival. Scores are the proportion of gender gap that has been closed, and range from 0.51 to 0.86.

The Women’s Economic Rights (WECON) and Women’s Political Rights (WOPOL) measures, from the CIRI Human Rights Data Project [37], include information for 202 countries for the years 1981–2011. WECON indicates the extensiveness of laws concerning women’s economic rights, such as equal pay for equal work, right to free choice of profession without a husband or male relative’s consent, and right to be free of sexual harassment, and how effectively the government enforces these laws. A WECON score of 0 indicates that there are no legal economic rights for women and that systematic gender-based discrimination may have been built into the law. The maximum score of 3 indicates that the law guarantees all or nearly all of women’s economic rights and the government fully enforces these laws.

WOPOL addresses comprehensiveness of laws pertaining to women’s political rights and how well the government enforces these laws. This measure includes: the rights to vote, the right to run for political office, the right to hold elected and appointed government positions, the right to join political parties and the right to petition government officials. A score of 0 indicates that the law does not guarantee political rights for women for that given year, and that there are laws that restrict women’s participation in the political arena and other areas of public life. The maximum score of 3 indicates that women’s political rights were guaranteed by law and practice, and that women hold more than 30% of seats the in national legislature and in other high ranking political offices. 

Note that for all four measures, higher values indicate less gender inequality.

#### Total fertility rate

TFR data came from the World Population Prospects 2015 revision [2]. TFR was expressed as the average number of children a hypothetical cohort of women would have if they were subject to the fertility rates of a given period and if they were not subject to mortality. We used the TFR values for 2005–2010 (which we term TFR 2010) to align with 2008 and 2012 smoking prevalence data.

An initial exploratory analysis found that some countries with low TFR also had low female smoking prevalence. These countries were mostly recently developed countries whose TFR values were substantially higher in the recent past. Because studies have shown that grandmothers, mothers and mothers-in-law play key roles in advising women on pregnancy diet, we hypothesized that the TFR rates experienced by these older women might influence their advice to younger pregnant women. Additionally, many women whose reproductive patterns contributed to earlier TFR estimates are still alive and contributing to smoking patterns in 2012. We therefore also used TFR values from approximately one generation earlier, 1975–80 (which we term TFR 1980), when many more countries had a TFR >4.

### Control variables

#### Gini coefficient

The Gini coefficient is a general measure of income inequality that ranges from 0, indicating complete equality, to 1, indicating complete inequality. Hitchman and Fong use the Gini coefficient from the UNDP’s Human development report 2009 [35] as a control variable so as to examine the unique impact of the GEM (female inequality).

#### Gross national income

To control for level of economic development, which is tied to progression through the tobacco epidemic, Hitchman and Fong used the log of GNI per capita for 2008 from the World Bank, expressed in international or purchasing power parity dollars. We added data from 2012 [38] to match our 2012 smoking data.

### Statistical analysis

All statistics were computed using R version 3.1.0 (2014-04-10) [39] with the following packages: nlme [40] to fit linear mixed effects models; effects [41] for effect plots; stargazer [42] to format statistical tables and knitr [43] to format the document. All analyses were of countries with complete data for the variables in that analysis (i.e. there was no imputation of missing data).

Residuals of all multiple regression models were plotted to assess model fit. Models of log 2010 smoking prevalence (see below) exhibited heteroscedasticity, which was addressed by using a variance function (varPower from the nlme package) [40]: $s^{2}(x)=|x{|}^{2t}$, where t is the variance function coefficient fit by the model and x was the fitted values.

## RESULTS

Summary statistics are presented in Table 2.

**Table 2.** Summary statistics for study variables

	*N*	Min	Max	Median	Mean	SD
**Outcome variables**						
Female smoking prevalence 2008 (%)	131	0.20	53.00	7.90	12.00	11.00
Male smoking prevalence 2008 (%)	130	5.30	70.00	32.00	33.00	14.00
Female smoking prevalence 2012 (%)	187	0.60	35.00	4.50	8.60	8.30
Male smoking prevalence 2012 (%)	187	5.00	61.00	25.00	27.00	12.00
**Explanatory variables**						
GEM	109	0.14	0.91	0.58	0.59	0.16
GGGI	142	0.51	0.86	0.69	0.69	0.06
WOPOL	182	1.00	3.00	2.00	2.10	0.51
WECON	182	0.00	3.00	1.00	1.30	0.90
TFR (2005–10)	184	1.20	7.70	2.50	3.00	1.60
TFR (1975–80)	184	1.50	8.50	5.20	4.80	2.00
**Control variables**						
Gini coefficient	142	25.00	74.00	40.00	41.00	9.10
Log 10 GNI per capita 2008	166	2.40	4.80	3.80	3.80	0.55
Log 10 GNI per capita 2012	178	2.80	5.10	4.00	4.00	0.52

*N* is the number of countries.

### Bivariate correlations

**Figure 2.**
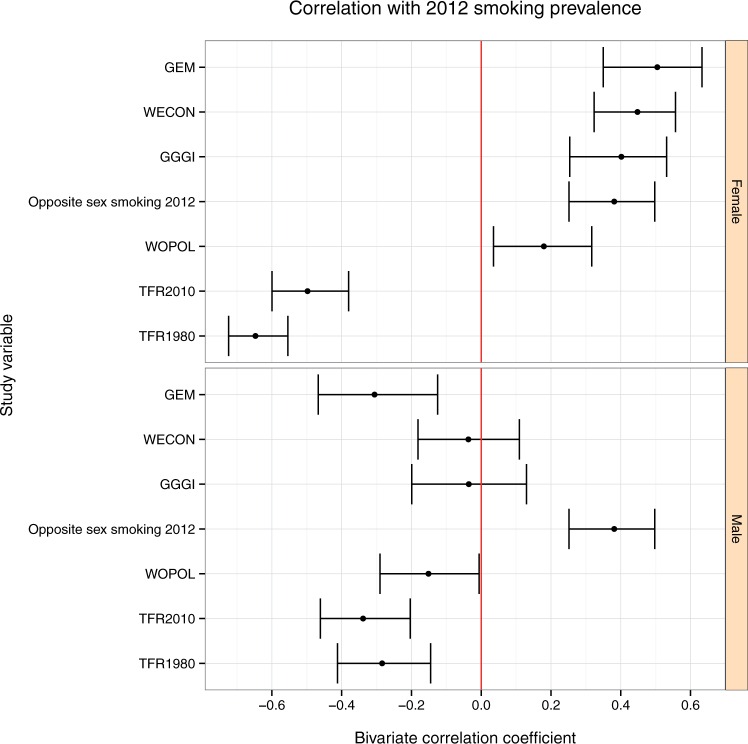
Bivariate correlations between study variables and 2012 smoking prevalence for men and women (sorted by correlation with female smoking). Bars are 95% CI. See text for data sources

Cross-national female and male smoking prevalence were only modestly correlated in both the 2008 data ($r=0.43,P=2.9\times 10^{-7}$) and 2012 data ($r=0.38,P=7.3\times 10^{-8}$), and these values were smaller, in absolute magnitude, than the correlation between female smoking prevalence and several of our explanatory variables, see Fig. 2.

### Reanalysis of data

**Figure 3.**
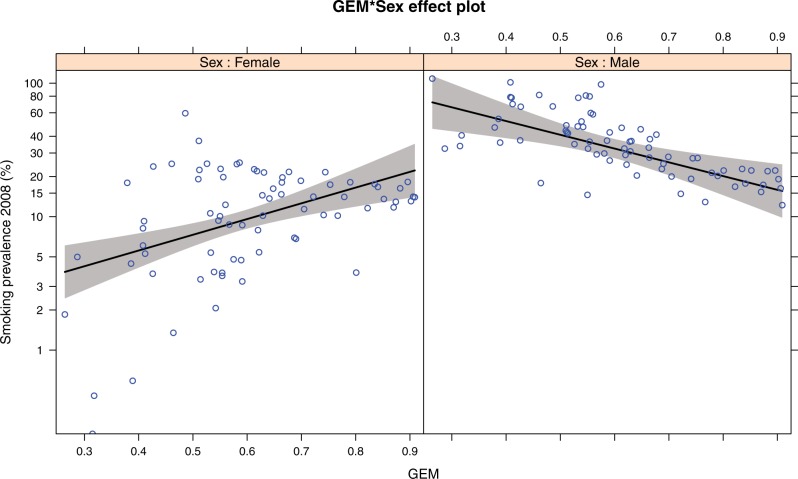
Effect plots of the association of GEM and 2008 male and female smoking prevalence, controlling for the Gini coefficient and log GNI. Each point is one country. Plotted at median values of the Gini and GNI, see Table 3, Model 2

Using the data from Ref. [6], we first replicated their model of the GSR (Table 3, Model 1). The modest correlation of female and male smoking prevalences suggested, however, that these might be influenced differently by potential explanatory variables. Hence, we examined the associations of potential explanatory variables on female and male prevalence separately, rather than on their ratio. We fit a model of log smoking prevalence with sex (male, female) entered as a dichotomous variable, along with its interaction with GEM. Because each country had two prevalence values (male and female), we fit a mixed effects model with country as the grouping variable. This model found that GEM had a significant positive association with female smoking prevalence and a significant negative association with male smoking prevalence (see Fig. 3 and Table 3, Model 2).

**Table 3.** Regression models of the association of GEM with male and female smoking prevalence using data from Hitchman and Fong [6]

	Dependent variable:		
	GSR	log10 (Prevalence2008)	
	OLS	Linear	
		Mixed effects	
	Model 1	Model 2	Model 3
Sex male		0.557^***^	0.570^***^
		(0.043)	(0.057)
Scale (GEM)	0.142^***^	0.189^***^	0.220^***^
	(0.037)	(0.047)	(0.042)
Scale (TFR2010)			−0.235^**^
			(0.079)
Scale (GINI)	0.022	0.027	0.003
	(0.026)	(0.029)	(0.017)
log10 (GNI2008)	0.211*	0.459^***^	0.064
	(0.083)	(0.092)	(0.068)
Sex male:scale (GEM)		−0.354^***^	−0.297^***^
		(0.043)	(0.041)
Sex male:scale (TFR2010)			0.110
			(0.080)
Constant	−0.427	−0.878*	0.704*
	(0.329)	(0.372)	(0.279)
Observations	74	149	149
*R*^2^	0.538		
Adjusted *R*^2^	0.519		
Log Likelihood		−40.330	−2.351
Akaike Inf. Crit.		96.660	28.703
Bayesian Inf. Crit.		120.363	64.088
Residual Std. Error	0.210 (df = 70)		
F Statistic	27.213^***^ (df = 3; 70)		

Complete data were available for 74 countries, except that Honduras male smoking was missing. Model 1 is the original model of the GSR in Hitchman and Fong. Model 2 predicts log smoking prevalence as a function of GEM interacting with Sex, controlling for GINI and log GNI. Model 3 adds TFR to model 2, which improves fit by according to AIC and BIC. Variables in interactions were centered and scaled by their standard deviations. SE in parentheses.

* *P* < 0.05, ^**^P < 0.01, ^***^P < 0.001.

### Adding TFR to the model

We then added TFR to our modified version of the Hitchman and Fong model. In this model (Table 3, Model 3), which improved fit according to the Akaike information criterion (AIC) and the Bayesian information criterion (BIC), GEM had a significant positive association with female smoking prevalence and a significant negative association with male prevalence. A 1 SD increase in GEM increased mean female smoking prevalence by a factor of 1.66, adjusting for covariates. TFR had a strong significant negative association with female prevalence, and a smaller negative association with male prevalence. A 1 SD increase in TFR decreased mean female smoking prevalence by a factor of 0.58, adjusting for covariates. After adding TFR, log GNI was no longer a significant predictor.

### 2012 Smoking prevalence

Our analysis of the 2012 smoking prevalence data parallels that of the 2008 data, except that we drop the Gini coefficient from the models because values for 39 countries were missing (its effect in 2008 models was small and not significant; Table 3).

Smoking prevalence within geographical regions is correlated (Supplementary Fig. S3), which can result in misleading estimates and *P*-values (Galton’s problem; [44]). In addition, compared to rest of the world, sub-Saharan Africa has both low female smoking prevalence and high TFR. Hence, any relationship between TFR and female smoking could be largely due to the effect of sub-Saharan Africa. We address these related problems in two ways.

#### Random effect for geographic region

First, the Global Burden of Disease project groups countries based on epidemiological similarity and geographic contiguity [45]. We compared models with a random effect for the 21 Global Burden of Disease regions to those without using likelihood ratio tests [40]. Models with a random effect for geographical region had significantly better fit than those without, so we report models with the region random effect.

The gender inequality measures were significant positive predictors of female smoking prevalence, and significant negative predictors of male smoking prevalence in all four models. A 1 SD increase in gender equality was associated with an increase in mean female smoking prevalence by factors ranging from 1.2 to 1.41, adjusting for covariates.

**Figure 4.**
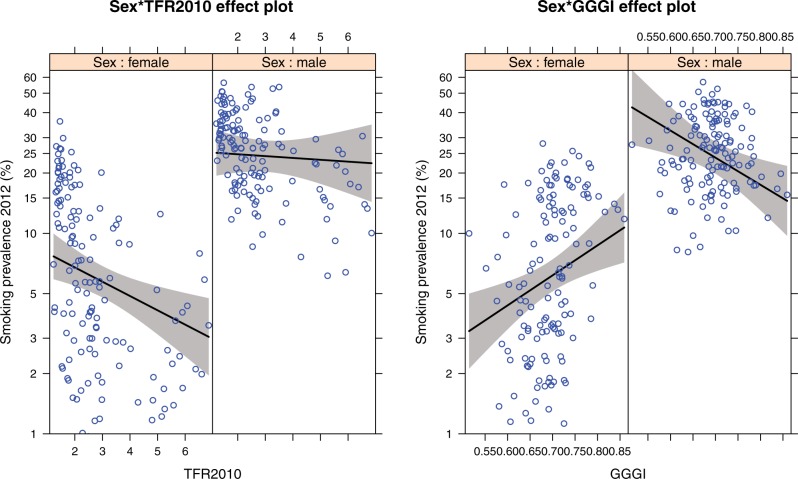
Effect plots of the associations of TFR and GGGI on 2012 male and female smoking prevalence, controlling for the GINI index and log GNI. Each point is one country. Plotted at median values of GINI and GNI, see Table 4, Model 2

TFR was a significant negative predictor of female smoking in all four models; there was also a significant interaction of TFR and sex in all models, such that TFR had a smaller, negative association with male smoking prevalence. A 1 SD increase in TFR was associated with a decrease in mean female smoking prevalence by factors ranging from 0.77 to 0.68, adjusting for covariates. Log GNI was not significant in any model, see Table 4 and Fig. 4 for depiction of one model (GGGI).

**Table 4.** Regression models of the associations of TFR, GEM, GGGI, WOPOL and WECON with log 2012 male and female smoking prevalence, with a random effect for the Global Burden of Disease regions

	Dependent variable:			
	log10 (Prevalence2012)			
	Model 1	Model 2	Model 3	Model 4
Sex male	0.570^***^	0.630^***^	0.653^***^	0.664^***^
	(0.037)	(0.031)	(0.029)	(0.029)
Scale (GEM)	0.149^***^			
	(0.033)			
Scale (GGGI)		0.089^**^		
		(0.029)		
Scale (WOPOL)			0.080^**^	
			(0.025)	
Scale (WECON)				0.107^***^
				(0.028)
Scale (TFR2010)	−0.168^**^	−0.113^**^	−0.165^***^	−0.126^**^
	(0.052)	(0.037)	(0.039)	(0.042)
log10 (GNI2012)	−0.032	0.008	−0.008	−0.002
	(0.062)	(0.064)	(0.052)	(0.050)
Sex male:scale (GEM)	−0.265^***^			
	(0.031)			
Sex male:scale (GGGI)		−0.169^***^		
		(0.033)		
Sex male:scale (WOPOL)			−0.103^***^	
			(0.027)	
Sex male:scale (WECON)				−0.196^***^
				(0.030)
Sex male:scale (TFR2010)	0.119*	0.098^**^	0.186^***^	0.103^**^
	(0.052)	(0.033)	(0.034)	(0.038)
Constant	0.978^***^	0.724^**^	0.770^***^	0.738^***^
	(0.260)	(0.260)	(0.211)	(0.202)
Observations	214	278	346	346
Log Likelihood	17.438	−31.887	−36.692	−24.451
Akaike Inf. Crit.	−12.876	83.774	95.383	70.903
Bayesian Inf. Crit.	24.150	120.050	137.694	113.214

Variables in interactions were centered and scaled by their standard deviations.

* *P*<0.05, ^**^P<0.01, ^***^P<0.001.

We then fit identical models of 2012 smoking prevalence that substituted TFR 1980 for TFR 2010. According to AIC, each TFR 1980 model outperformed the matching model with TFR 2010. A 1 SD increase in TFR 1980 was associated with a decreased mean 2012 female smoking prevalence by factors ranging from 0.62 to 0.53, adjusting for covariates, see Supplementary Table S1. 

Log GNI was a not a significant predictor of smoking prevalence in any model.

#### Models excluding countries in sub-Saharan Africa

Second, to check if our results were largely a consequence of patterns seen in sub-Saharan Africa, we fit a set of models that excluded those countries.

Models of smoking that excluded countries in sub-Saharan Africa found similar significant negative associations with 2010 TFR (Supplementary Table S2), albeit based primarily on the small number of non-African countries with high TFR. Versions of these models with 1980 TFR showed better fit to 2012 smoking prevalence because in 1980 many non sub-Saharan African countries had high TFR; according to AIC, each of 1980 models outperformed the matching 2010 model (Supplementary Table S2).

#### Smoking prevalence pre- vs post-menopause (2012)

**Figure 5.**
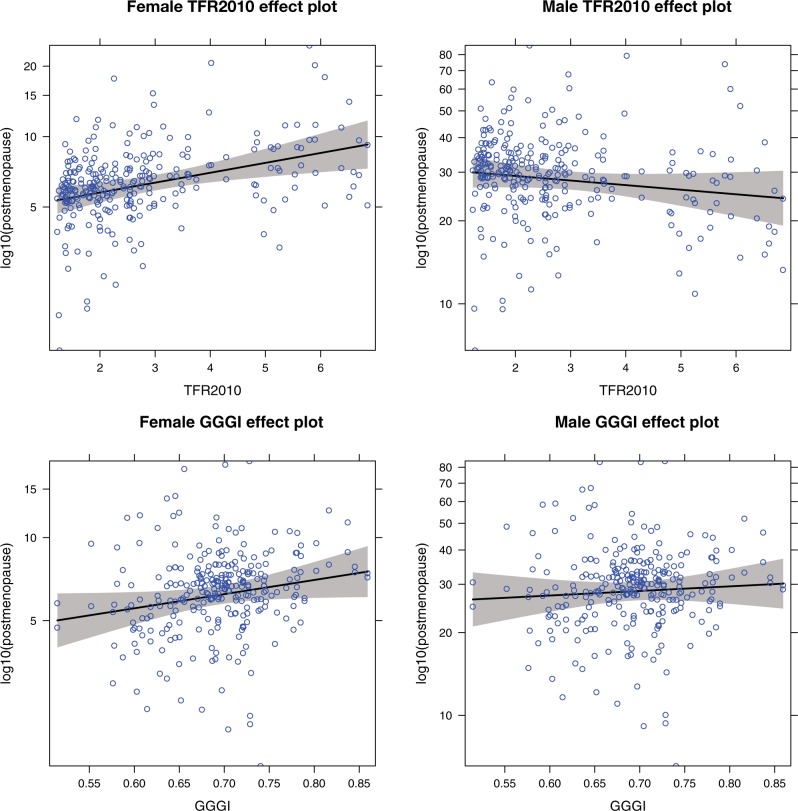
Effect plots of the associations of 2010 TFR and GGGI on log postmenopausal 2012 smoking prevalence, controlling for log pre-menopausal prevalence and log GNI. Each point is one country. Left: female smoking prevalence. Right: male smoking prevalence. Plotted at median values of GNI, see Table 5, Model 2

Two of four gender inequality measures (GEM, WOPOL) were significant positive predictors of 2012 postmenopause female smoking prevalence, controlling for premenopause smoking prevalence, log GNI and TFR, which was contrary to the predicted negative association. TFR was a significant positive predictor of postmenopause female smoking prevalence across all four models, as predicted, with an associated increase in older women’s smoking prevalence by factors ranging from 1.11 to 1.21, relative to younger women (adjusting for covariates). There was a significant interaction of TFR with sex across all four models, such that the slope of TFR on older male smoking was close to zero, as predicted. Log GNI had a negative association with postmenopause smoking, which was significant in three of four models, see Table 5 and Fig. 5 for depiction of one model GGGI. Models fit using 1980 TFR had somewhat larger coefficients for TFR than those fit with 2010 TFR (Supplementary Table S3).

**Table 5.** Regression models of log post-menopause smoking prevalence (2012) as a function of 2010 TFR and four measure of gender inequality, controlling for log pre-menopause prevalence and log GNI, and with a random effect for GBD regions

	Dependent variable:			
	log10 (postmenopause)			
	Model 1	Model 2	Model 3	Model 4
log10 (premenopause)	0.789^***^	0.742^***^	0.796^***^	0.790^***^
	(0.037)	(0.027)	(0.026)	(0.027)
Sex male	0.054*	0.052*	0.026	0.028
	(0.022)	(0.026)	(0.020)	(0.021)
Scale (TFR2010)	0.083^***^	0.067^**^	0.047^**^	0.045*
	(0.020)	(0.021)	(0.017)	(0.019)
Scale (GEM)	0.047^**^			
	(0.017)			
Scale (GGGI)		0.030		
		(0.015)		
Scale (WOPOL)			0.040^***^	
			(0.011)	
Scale (WECON)				−0.002
				(0.014)
log10 (GNI2012)	−0.051	−0.079*	−0.069^**^	−0.059*
	(0.033)	(0.034)	(0.023)	(0.024)
Sex male:scale (TFR2010)	−0.081^***^	−0.093^***^	−0.075^***^	−0.081^***^
	(0.019)	(0.019)	(0.015)	(0.018)
Sex male:scale (GEM)	−0.053^**^			
	(0.018)			
Sex male:scale (GGGI)		−0.021		
		(0.018)		
Sex male:scale (WOPOL)			−0.022	
			(0.013)	
Sex male:scale (WECON)				−0.017
				(0.016)
Constant	0.463^**^	0.636^***^	0.534^***^	0.503^***^
	(0.144)	(0.139)	(0.096)	(0.099)
Observations	214	278	346	346
Log Likelihood	127.396	113.170	188.666	182.836
Akaike Inf. Crit.	−230.792	−204.339	−353.333	−341.672
Bayesian Inf. Crit.	−190.858	−164.757	−307.456	−295.795

Variables in interactions were centered and scaled by their standard deviations. Data from Ng *et al.* (2014).

* *P*<0.05, ^**^P<0.01, ^***^P<0.001.

## DISCUSSION

Our most important finding is that TFR had a significant negative association with female smoking prevalence in all models, and a smaller negative association with male smoking prevalence, even after controlling for multiple indices of gender inequality and GNI (Tables 3 and 4), and even if countries in sub-Saharan Africa were excluded (Supplementary Table S2). The sex difference (interaction term) was in the predicted direction in all models, and statistically significant in all but Model 3 of Table 2. These results, along with the finding that among postmenopausal women TFR was a significant positive predictor of smoking prevalence, but among older men was a significantly smaller predictor of smoking prevalence that was close to zero (controlling for premenopausal/younger adult smoking prevalence; Table 5), supports the hypothesis that acute tobacco toxicity has a particularly important influence on female smoking behaviors, especially among younger adult women in countries with high TFR.

Reanalysis of the data from Ref. [6] revealed that, controlling for GINI and log GNI, GEM had a significant positive association with log female smoking prevalence that was approximately proportionate to its negative association with male prevalence (Table 3, Model 2). Analysis of four indices of gender inequality (GEM, GGGI, WECON, WOPOL) using smoking prevalence from a larger and more recent data set [1], found that each had a significant positive association with log transformed female smoking prevalence. These results support the gender inequality model. In addition, the interaction with sex was significant for all four indices, each resulting in an unexpected negative association with male smoking prevalence (Table 4). This negative relationship suggests gender inequality is correlated with tobacco control efforts, a hypothesis to test in future research.

The possible confound with tobacco control could also explain the negative relationship between TFR and male smoking. Alternatively, smoking has a negative association with male fertility [46]. Cholinergic signaling plays a role in testes cells [47], in particular the contraction of smooth muscle in the testicular capsule that propels sperm from the seminiferous tubules to the epididymis; contractile dysfunctions in the testicular capsule are implicated in male infertility [48]. This and other testicular functions of acetylcholine might be disrupted by nicotine, which is a cholinergic toxin. It is therefore possible that, in countries with high TFR, men avoid toxic plant compounds too (albeit less so than women).

Finally, two of four gender inequality measures had a significant ‘positive’ association with postmenopausal smoking prevalence, which contradicts the prediction from the gender inequality model that, in countries with greater gender inequality, there would be a greater difference in women’s smoking prevalence pre- vs post-menopause.

### Limitations

We caution that our analysis is based on aggregated cross-sectional survey data from varied geographic, political and cultural contexts. Such data cannot be used to infer causality, nor can it be assumed to be of consistent accuracy and may include studies with methodological biases. Cross-cultural surveys are vulnerable to both numerator (case finding) and denominator (population description) problems, particularly when local cultural issues are not factored into research design, e.g. Ref. [49]. For example, in certain cultural settings, gendered power imbalances may make it more difficult for women to discuss or admit to personal drug-use behaviors, and/or cultural traditions may prohibit women from using certain types of drugs, e.g. Ref. [50]. The main concern for our own study is whether or not the aggregated data may include systematic undercounting of female smokers in low-income vs high income settings.

Furthermore, to maintain comparability with Hitchman and Fong [6], we did not include additional control variables, such as the relative price of cigarettes and extent of tobacco control programs. Thus, future research may seek to mitigate potential cross-cultural case-finding bias using new meta-analysis data filtered by culturally sensitive research design, and should control for additional potentially confounding factors.

### Implications for public health

Research on substance abuse and addiction has focused heavily on reward signaling pathways. The perspective advocated here suggests that evolved toxin defense pathways might be effective targets of intervention to reduce drug consumption, perhaps especially in women of reproductive age. For example, there is increasing evidence that progesterone, a critical reproductive hormone, has a number of suppressive effects on female smoking [51]. Although these effects are attributed to progesterone’s interactions with brain reward circuitry, they might instead be due to progesterone’s activation of toxin defense mechanisms, as evidenced by progesterone’s up-regulation of P450 toxin metabolizing enzymes. Further research should determine the extent to which hormone changes in pregnancy activate toxin avoidance and defense mechanisms, and the extent to which such mechanisms deter tobacco use.

Taking into account our finding that both TFR and gender inequality are independently associated with female smoking prevalence, that TFR values from approximately one generation earlier outperform recent TFR values in our models, and that research on cultural transmission of pregnancy diet found that older female relatives advise younger pregnant women, we present the following theoretical synthesis. We propose that rather than restricting women’s access to valued rewarding psychoactive substances that they would otherwise consume, cultural norms serve to reinforce pregnant women’s innate aversion to substances that might harm their fetuses and nursing infants. In countries with high TFR and lower use of modern birth control, these norms might be especially effective at preventing female tobacco use.

If modern birth control and fertility reduction is responsible, in part, for increases in women’s smoking, then, paradoxically, it might be efficacious to integrate tobacco control into programs for family planning and provisioning of birth control, and not only pregnancy-related health care. In countries with high TFR, an emphasis on tobacco’s harmful effects on future fertility and early onset of menopause [52] might resonate especially strongly with both genders, as tobacco also has a negative impact on male fertility [46]. Future research should determine the extent to which warnings about harm to fertility deter tobacco use, especially in populations with high TFRs.

## Supplementary Material

Supplementary Data
