# Supplementary material for: The low prevalence of female smoking in the developing world: gender inequality or maternal adaptations for fetal protection?
Source: Evol Med Public Health. 2016 May 18;2016(1):195–211. doi: 10.1093/emph/eow013 (PMC4931906; doi:10.1093/emph/eow013)
Supplement: Supplementary Data [file supp_2016_1_195__index.html]

Supplementary Data 

# The low prevalence of female smoking in the developing world: gender inequality or maternal adaptations for fetal protection?

## Supplementary Data

files

- Supplementary Data - pdf file
