# Supplementary material for: The low prevalence of female smoking in the developing world: gender inequality or maternal adaptations for fetal protection?
Source: Evol Med Public Health. 2016 May 18;2016(1):195–211. doi: 10.1093/emph/eow013 (PMC4931906; doi:10.1093/emph/eow013)
Supplement: Supplementary Data [file supp_eow013_Supplementary_information.pdf]

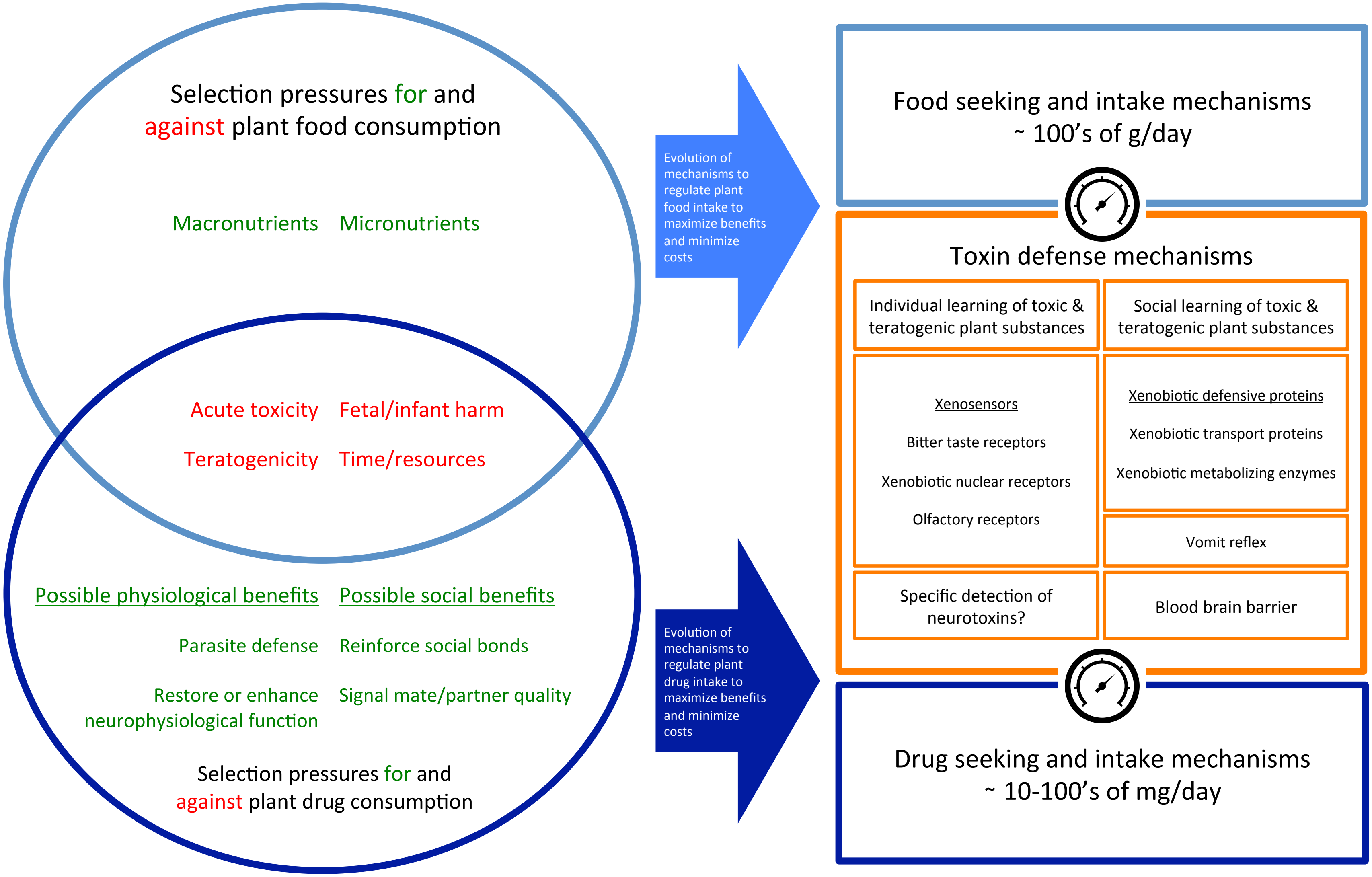

Figure S1: Theoretical model of the evolution of mechanisms to regulate food and plant drug intake so as to maximize benefits against costs

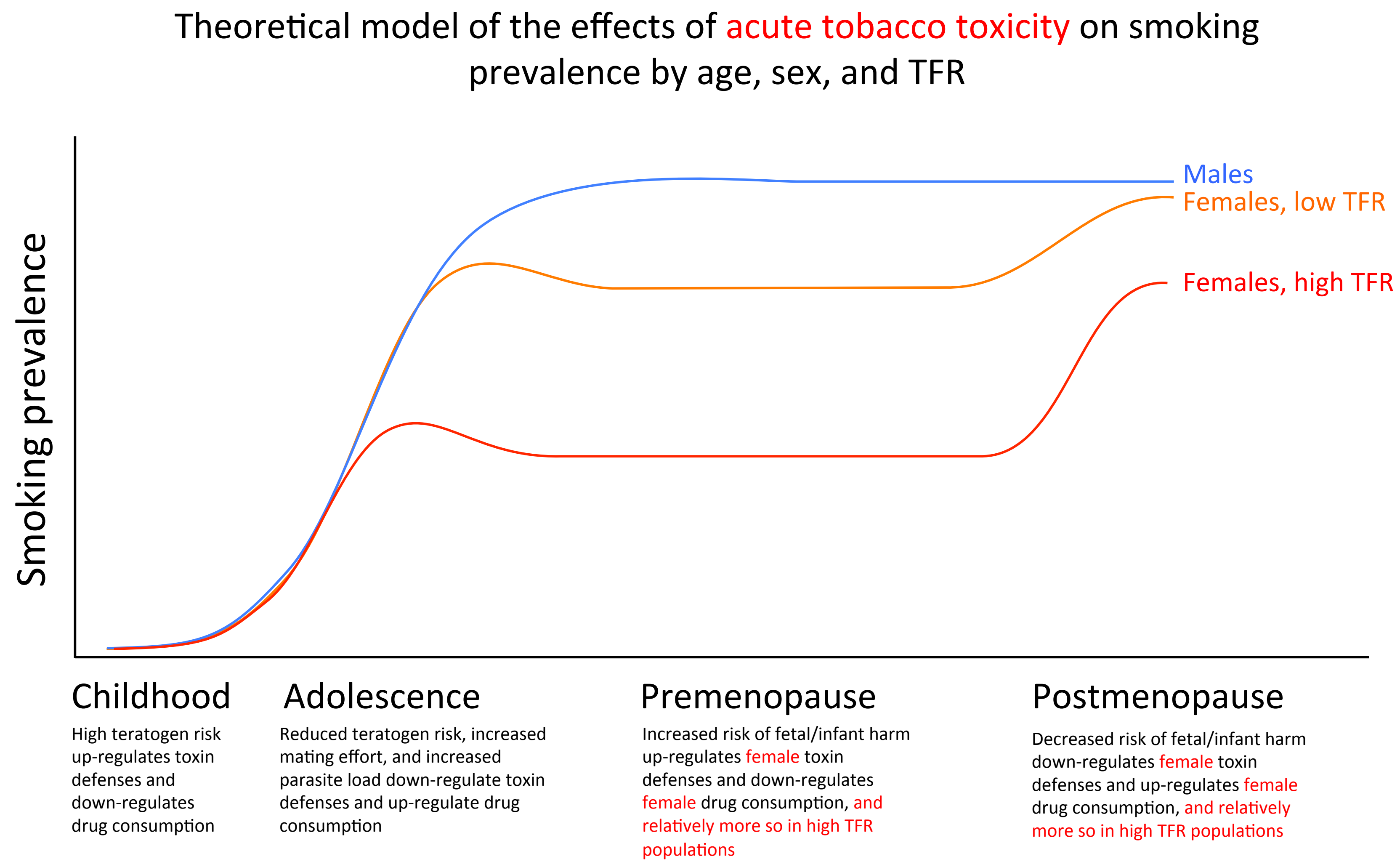

Figure S2: Predicted age and sex differences in smoking over the lifespan in countries with high vs. low total fertility rates (TFR)

Figure S3: Distribution of smoking prevalence for females and males within Global Burden of Disease super regions.

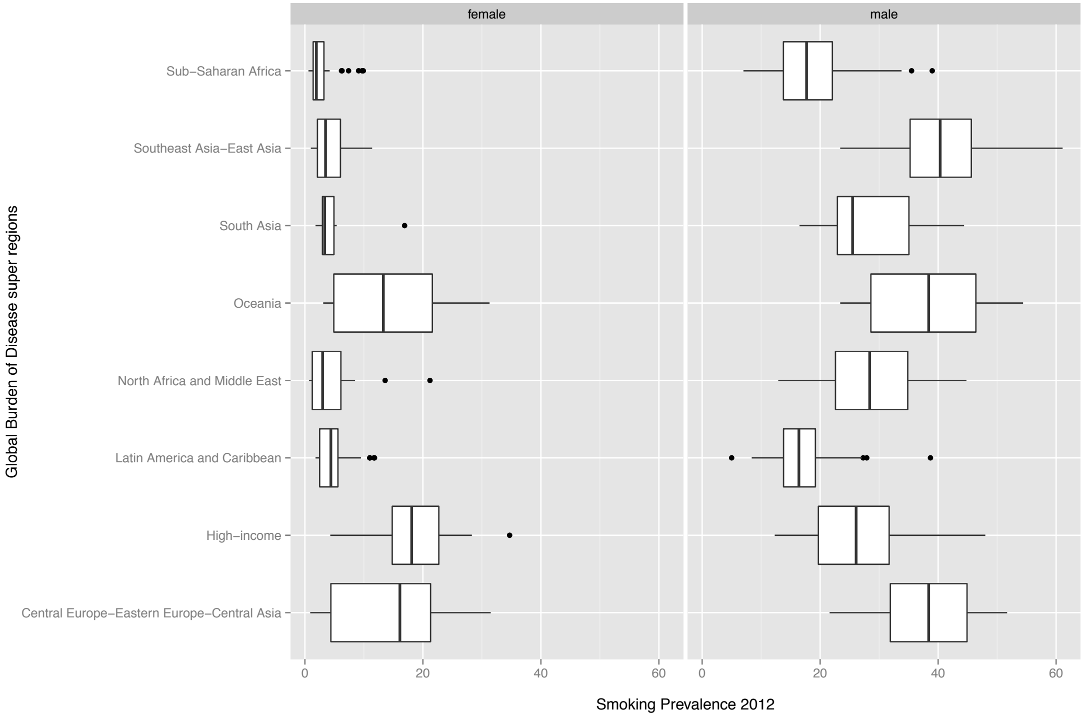

Table S1: Regression models of the associations of 1980 TFR on 2012 male and female smoking prevalence using data from Ng et al. (2014), controlling for GGGI, WOPOL, WECON, and with a random effect for the Global Burden of Disease regions. Variables in interactions were centered and scaled by their standard deviations.

	Dependent variable:			
	log10(Prevalence2012)			
	(1)	(2)	(3)	(4)
Sexmale	0.571***	0.643***	0.661***	0.664***
	(0.035)	(0.030)	(0.028)	(0.027)
scale(GEM)	0.092*			
	(0.036)			
scale(GGGI)		0.065*		
		(0.029)		
scale(WOPOL)			0.029	
			(0.024)	
scale(WECON)				0.057*
				(0.028)
scale(TFR1980)	-0.207***	-0.273***	-0.249***	-0.214***
	(0.048)	(0.038)	(0.035)	(0.036)
log10(GNI2012)	-0.028	-0.057	-0.048	-0.033
	(0.059)	(0.052)	(0.047)	(0.047)
Sexmale:scale(GEM)	-0.213***			
	(0.037)			
Sexmale:scale(GGGI)		-0.116***		
		(0.033)		
Sexmale:scale(WOPOL)			-0.043	
			(0.027)	
Sexmale:scale(WECON)				-0.119***
				(0.031)
Sexmale:scale(TFR1980)	0.142**	0.222***	0.244***	0.187***
	(0.046)	(0.035)	(0.028)	(0.033)
Constant	0.959***	0.973***	0.920***	0.857***
	(0.248)	(0.214)	(0.192)	(0.190)
Observations	214	278	346	346
Log Likelihood	19.077	1.726	-12.109	-5.273
Akaike Inf. Crit.	-16.154	18.549	46.218	32.547
Bayesian Inf. Crit.	20.871	58.453	88.529	74.857
Note:		*p<.05; **p<.01; ***p<0.001		

Table S2: Non Sub-Saharan African countries. Regression models of the association of 2010 TFR (models 1-4) and 1980 TFR (models 5-8) on 2012 male and female smoking prevalence using data from Ng et al. (2014), controlling for GGGI, WOPOL, WECON, and with a random effect for the Global Burden of Disease regions, but excluding countries in sub-Saharan Africa. Variables in interactions were centered and scaled by their standard deviations.

	Dependent variable:							
	log10(Prevalence2012)							
	(1)	(2)	(3)	(4)	(5)	(6)	(7)	(8)
Sexmale	0.536***	0.572***	0.642***	0.592***	0.540***	0.572***	0.598***	0.590***
	(0.038)	(0.035)	(0.039)	(0.035)	(0.037)	(0.034)	(0.035)	(0.033)
scale(GEM)	0.151***				0.097*			
	(0.034)				(0.038)			
scale(GGGI)		0.095**				0.040		
		(0.034)				(0.034)		
scale(WOPOL)			0.119***				0.014	
			(0.031)				(0.031)	
scale(WECON)				0.152***				0.080*
				(0.030)				(0.031)
scale(TFR2010)	-0.131**	-0.067*	-0.243***	-0.117**				
	(0.044)	(0.033)	(0.050)	(0.044)				
scale(TFR1980)					-0.195***	-0.213***	-0.280***	-0.212***
					(0.053)	(0.038)	(0.043)	(0.043)
log10(GNI2012)	-0.056	-0.033	-0.072	-0.053	-0.070	-0.084	-0.139*	-0.099
	(0.062)	(0.075)	(0.056)	(0.051)	(0.063)	(0.069)	(0.056)	(0.054)
Sexmale:scale(GEM)	-0.263***				-0.211***			
	(0.032)				(0.039)			
Sexmale:scale(GGGI)		-0.198***				-0.114**		
		(0.039)				(0.043)		
Sexmale:scale(WOPOL)			-0.161***				-0.046	
			(0.034)				(0.034)	
Sexmale:scale(WECON)				-0.235***				-0.153***
				(0.031)				(0.034)
Sexmale:scale(TFR2010)	0.101*	0.080*	0.263***	0.110*				
	(0.044)	(0.039)	(0.048)	(0.043)				
Sexmale:scale(TFR1980)					0.142**	0.189***	0.263***	0.181***
					(0.052)	(0.043)	(0.039)	(0.041)
Constant	1.121***	0.983**	1.082***	1.053***	1.175***	1.194***	1.402***	1.244***
	(0.264)	(0.319)	(0.236)	(0.217)	(0.268)	(0.289)	(0.235)	(0.226)
Observations	194	220	258	258	194	220	258	258
Log Likelihood	25.674	-26.357	-18.710	2.474	23.386	-14.235	-2.165	11.406
Akaike Inf. Crit.	-29.348	72.713	59.421	17.052	-24.772	48.470	26.331	-0.811
Bayesian Inf. Crit.	6.599	106.649	98.503	56.135	11.175	82.406	65.413	38.272
Note:								*p<.05; **p<.01; ***p<0.001

Table S3: Regression models of log post-menopause smoking prevalence (2012) as a function of 1980 TFR and four measure of gender inequality, controlling for log pre-menopause prevalence and log GNI, and with a random effect for GBD regions. Variables in interactions were centered and scaled by their standard deviations. Data from Ng et al. (2014).

	Dependent variable:			
	log10(postmenopause)			
	(1)	(2)	(3)	(4)
log10(premenopause)	0.856***	0.786***	0.847***	0.823***
	(0.038)	(0.030)	(0.026)	(0.026)
Sexmale	0.010	0.019	-0.009	0.007
	(0.023)	(0.028)	(0.021)	(0.021)
scale(TFR1980)	0.123***	0.103***	0.087***	0.063***
	(0.023)	(0.021)	(0.016)	(0.018)
scale(GEM)	0.079***			
	(0.018)			
scale(GGGI)		0.047**		
		(0.015)		
scale(WOPOL)			0.051***	
			(0.011)	
scale(WECON)				0.005
				(0.014)
log10(GNI2012)	-0.058*	-0.055*	-0.020	-0.027
	(0.027)	(0.027)	(0.019)	(0.020)
Sexmale:scale(TFR1980)	-0.122***	-0.109***	-0.083***	-0.074***
	(0.022)	(0.022)	(0.015)	(0.018)
Sexmale:scale(GEM)	-0.078***			
	(0.020)			
Sexmale:scale(GGGI)		-0.033		
		(0.020)		
Sexmale:scale(WOPOL)			-0.031*	
			(0.013)	
Sexmale:scale(WECON)				-0.013
				(0.017)
Constant	0.441***	0.512***	0.305***	0.353***
	(0.121)	(0.111)	(0.081)	(0.083)
Observations	214	278	346	346
Log Likelihood	131.416	112.062	186.033	174.800
Akaike Inf. Crit.	-240.831	-204.124	-350.065	-327.599
Bayesian Inf. Crit.	-204.225	-168.140	-308.012	-285.546
Note:				*p**p***p<0.001
